# Imagining roles for epigenetics in health promotion research

**DOI:** 10.1007/s10865-016-9764-4

**Published:** 2016-07-13

**Authors:** Colleen M. McBride, Laura M. Koehly

**Affiliations:** 1 Department of Behavioral Sciences and Health Education, Rollins School of Public Health, Emory University, 1518 Clifton Rd. NE, GCR 564, Atlanta, GA 30322 USA; 2 National Human Genome Research Institute, Bethesda, MD USA

**Keywords:** Health promotion, Epigenetics, Interventions, Health behavior change

## Abstract

Discoveries from the Human Genome Project have invigorated discussions of epigenetic effects—modifiable chemical processes that influence DNA’s ability to give instructions to turn gene expression on or off—on health outcomes. We suggest three domains in which new understandings of epigenetics could inform innovations in health promotion research: (1) increase the motivational potency of health communications (e.g., explaining individual differences in health outcomes to interrupt optimistic biases about health exposures); (2) illuminate new approaches to targeted and tailored health promotion interventions (e.g., relapse prevention targeted to epigenetic responses to intervention participation); and (3) inform more sensitive measures of intervention impact, (e.g., replace or augment self-reported adherence). We suggest a three-step process for using epigenetics in health promotion research that emphasizes integrating epigenetic mechanisms into conceptual model development that then informs selection of intervention approaches and outcomes. Lastly, we pose examples of relevant scientific questions worth exploring.

## Introduction

Over the last two decades, discoveries from the Human Genome Project (HGP) and visions for applying genomics in everyday medical care (aka precision medicine) have invigorated discussions of epigenetics (Collins & Varmus, 2015). Findings that humans have considerably fewer genes than anticipated have supported notions that biological processes in addition to the genome—epigenetic mechanisms—must be influencing gene expression and observed variation in traits and health outcomes (Claverie, 2005). Further, there is evidence that the majority of common genetic variants (i.e., single nucleotide polymorphisms or SNPs) are of low penetrance and do not directly result in observable traits (Wild, 2005). Indeed, most of these variants appear to influence disease risk only in the presence of an environmental exposure that prompts epigenetic mechanisms and gene expression (Wild, 2005). Such emerging understandings likely will position epigenetics front and center in future discussions of genomics and health promotion research.

In this report, we consider where these new understandings have the potential to add value and foster innovation in the development and evaluation of health promotion interventions. We suggest three domains in which epigenetic mechanisms could inform such innovations: (1) updates in health risk information that could improve the motivational potency of health communications (e.g., explaining individual differences in health outcomes to interrupt optimistic biases about risk exposures); (2) illuminate new approaches to targeted and tailored interventions (e.g., relapse prevention targeted to epigenetic response to intervention participation); and (3) inform more sensitive measures of intervention impact (e.g., replace or augment self-reported adherence). Additionally, we discuss feasible ways to use epigenetics in health promotion interventions and related research methods, and provide practical “how to” steps for getting there. But, first, we go under the hood with a technical overview of epigenetic concepts that could inform these innovations.

## Technical overview of epigenetics

Epigenetic mechanisms are the chemical processes that influence the ability of deoxyribonucleic acid (DNA) to give instructions (i.e., whether and how genes are expressed) and influence whether phenotypes associated with gene variants become manifest physically or clinically (Feinberg, 2013). Environmental exposures are thought to prompt these chemical processes. Consider, for example, research showing that monozygotic twins who are genetically identical at birth develop different physical characteristics as they age (Fraga et al., 2005). These differences are thought to arise from the accumulation of epigenetic responses to increasingly divergent environmental exposures that twins experience as they spend less of their lives together (Fraga et al., 2005). Thus, epigenetic processes are suggested to explain how environmental exposures impact health across the lifespan.

### Epigenetic processes

Epigenetic mechanisms are now understood to play a critical role in regulation of gene expression, allowing different cells to express different portions of the genome (Herceg et al., 2013). The processes governing gene expression are rooted in how DNA is stored. DNA strands, too long to fit neatly into the nucleus of cells, are wrapped in a dynamic and functional structure called chromatin. DNA is then wound around three dimensional protein structures called “histones” or spools of different types that offer specific storage services and perform somewhat different mechanical functions to manage cell processes (Lawrence et al., 2016). These histones have “tails” that act as receiving stations for a variety of modifications made to the genome that influence gene expression and function.

The best understood of these chemical reactions is methylation, a biochemical process in which a methyl group (i.e., a chemical compound) gets added to the histone tail (Bakulski & Fallin, 2014; Bjornsson et al., 2004; Feinberg, 2013). This methylation, in part, modifies the histone by strengthening the charge (i.e., the magnetic hold) of the spool such that the DNA is packed more tightly around the spool. This tight packing makes the DNA less accessible and restricts DNA from being read (i.e. transcribed), essentially “turning off” gene expression. Alternatively, demethylation neutralizes the charge of the histone spool, loosening the tension and allowing the DNA to be read more easily, essentially “turning on” gene expression. It is noteworthy that most genes in humans are methylated or “turned off”. Thus, exposures in the environment often function by turning on gene expression (i.e., demethylation) and prompting pathological processes such as cell proliferation, a process that characterizes many cancers (Feinberg, 2013; Herceg et al., 2013). Most important for health promotion interventions is that demethylation can be reversed, offering an opportunity to reverse the negative impact of environmental exposures (Godfrey et al., 2007).

Methylation processes tend to occur in “CpG Islands” that is at locations in the genome where there are long repeated sequences of bases of a cytosine nucleotide or “C” located next to a guanine nucleotide or “G”; each of these C–G pairs are separated by one phosphate, hence the CpG denotation. In turn, CpG islands tend to be near sites of human gene “promoters” (How Kit et al., 2012). These regions of the gene are typically not methylated, comprise looser bonds that increase their ability to be read, and are thought to be especially important in regulating gene expression.

As described above these epigenetic mechanisms are thought to be influenced by exposures that span from micro “in vivo” exposures to macro-level social influences. Indeed, Wild refers to this as the “exposome” that includes every exposure to which an individual has been subjected from conception to death (Wild, 2005).

### Conceptualizing the exposome

Early conceptualizations of epigenetics emphasized the toxicological role of exposures in damaging DNA (e.g., tobacco exposure), which in turn contributed to cancer and other disease etiology (Wild et al., 2013). Such conceptualizations have been broadened over time to give attention to social, behavioral and psychological factors that can influence epigenetic mechanisms and gene expression (Myers, 2009). These include but are not limited to social capital, education, financial status, health behaviors, and psychological stress. Additionally, these exposures can include social structural and cultural experiences that come, for example, from living in rural versus urban environments and the contexts of differing social norms (Gehlert et al., 2008; Juarez et al., 2014; Thayer & Kuzawa, 2011). More recently the built environment, a key factor implicated in health disparities, has been added as part of this “public health exposome” (Juarez et al., 2014).

Integrating epigenetics into health promotion interventions aligns well with the field’s growing emphasis on socio-ecological frameworks in which multiple levels of social and behavioral influences on health are considered as a set of “nested complexities” (Glass & McAtee, 2006). Epigenetics could aid in characterizing how these influences or exposures become embodied via their influence on gene expression (Essex et al., 2013; Krieger, 1999).

It is well known that epigenetic alterations accumulate with age (Sierra et al., 2015). However, understandings of epigenetic mechanisms also indicate that the timing and chronicity of exposure is very important. Thus, considering epigenetic mechanisms reifies suggestions that health promotion research take a lifespan approach (Uchino, 2009). For epigenetics, environmental responsivity is especially heightened at some periods of human development including perinatal, peri-pubertal, and for women, during the menopausal transition (Kanherkar et al., 2014). Some have suggested that health promotion interventions might best be targeted to these “windows of responsivity” when exposures may be particularly influential. Additionally, interventions could be developed for those who share profiles of risk exposure that occurred at periods of heightened responsivity (Burdge & Lillycrop, 2010). In this way, understandings of epigenetic mechanisms could offer new conceptualizations for considering levels of influence, as well as selection and timing of intervention approaches and outcomes.

### Measuring epigenetic processes

Currently, epigenetic processes are measured by using a variety of bioinformatics approaches to identify regions of the genome with a high density of CpG islands (Bakulski & Fallin, 2014; Shen & Waterland, 2007). Repetitive elements (REs) such as CpG islands are over- or under-represented in some areas of the genome. Typically, to measure epigenetic modifications, these areas of the genome are searched for regions characterized by: length (e.g., >500 base pairs), number of GC pairs (over 55 %), the ratio of observed to expected number of CpG repeats (>0.65) and the physical distance between neighboring CpG islands (How Kit et al., 2012; Shen & Waterland, 2007).

**Fig. 1:**
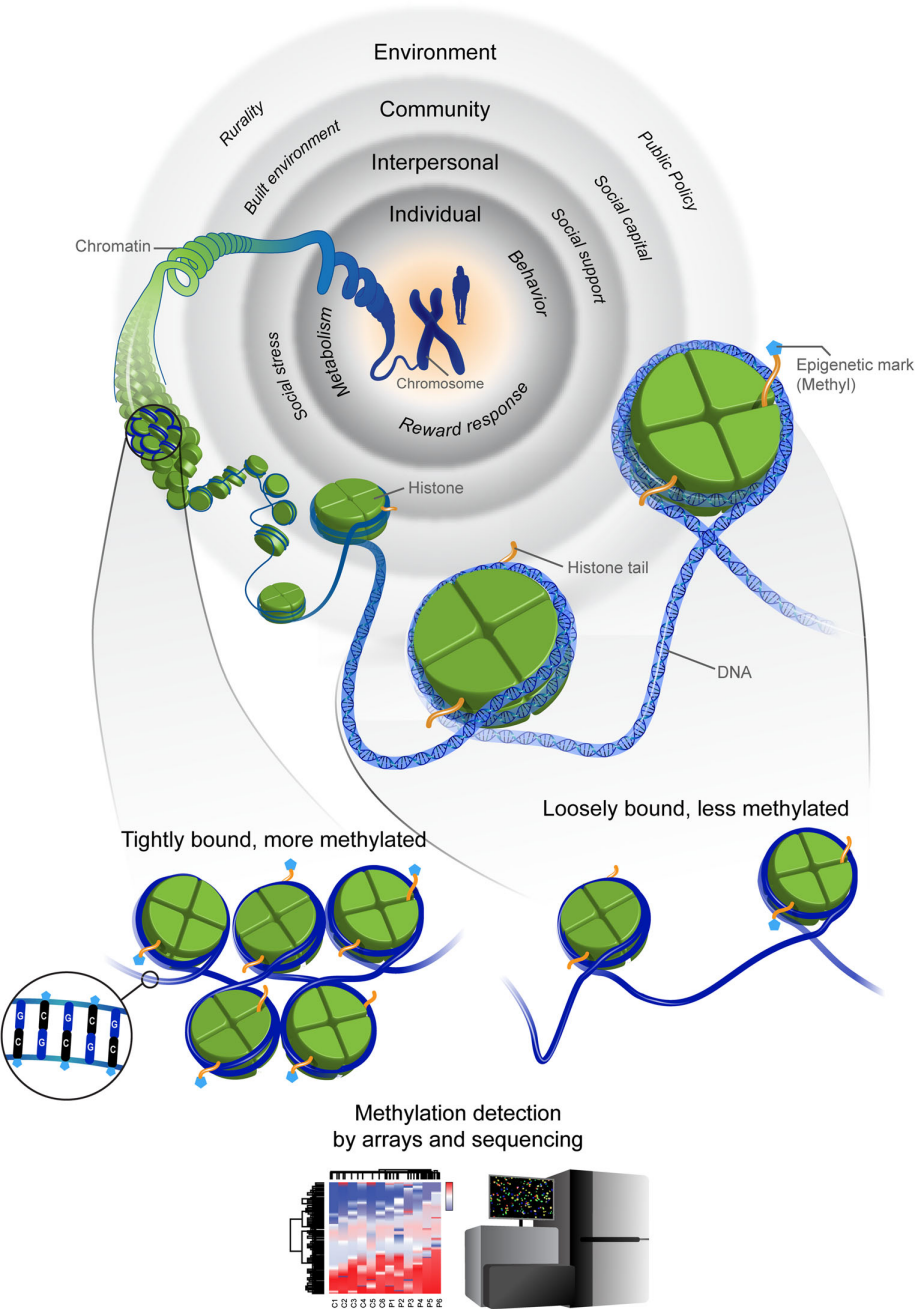
Conceptualizing the exposome and epigenetic processes. The *blue strands* of DNA are wrapped in a dynamic and functional structure called chromatin. As illustrated in the figure, the DNA is wound around histones. Histone tails receive modifications, or epigenetic marks, that turn on or turn off gene expression. One such modification is methylation, in which a methyl group, represented by the *blue pentagon*, attaches to the histone tail. DNA is wrapped more tightly around histones that are highly methylated, restricting accessibility of the DNA to be read for gene expression. Methylation of DNA occurs in areas of density in cytosine nucleotides and guanine nucleotides (CpG islands). Epigenetic processes can occur in response to nested levels of exposures depicted at the: individual, interpersonal, community, and environmental levels; each can influence epigenetic modifications independently or jointly. Epigenetic responses may result in more methylation that tightens the chromatin bond “turning off” gene expression; or, such processes may give rise to demethylation, resulting in loosely bound chromatin “turning on” gene expression. Thus, detecting the amount of methylation across the genome or within a particular gene using arrays or with sequencing technologies can provide evidence of epigenetic responses to a set of exposures (Color figure online)

Areas known to be rich in concentration of REs (e.g., CpG islands) can be interrogated to indicate global methylation, rapidly and relatively economically (How Kit et al., 2012; Shen & Waterland, 2007). Other approaches assess locus-specific methylation either for a candidate gene or genome-wide. These methods begin by focusing on specific genes identified from Genome Wide Association Studies (GWAS) (Shen & Waterland, 2007). GWAS studies are based on large numbers of cases and controls and can be used to identify genes that lie in biologically plausible pathways (e.g., inflammation, reward, metabolism) for a specified behavioral or health outcome. Such pathways also will inform the optimal timing of epigenetic assessments. Notably, epigenetic assessments will require study designs that include prospective and repeated measures designs in which individuals can serve as their own controls; study designs that characterize health promotion research (Bakulski & Fallin, 2014). Figure 1 provides a conceptual overview of the links between exposome-level factors and epigenetic mechanisms.

### Summary

There are three broad conclusions to take away from this technical overview of epigenetics. Firstly, epigenetic mechanisms are influenced by a broad array of environmental exposures, amenable to interventions, and reversible (Godfrey et al., 2007; Loi et al., 2013). Second, there are systematic methods for assessing epigenetic modifications to the genome that are: increasingly affordable, and derivable from biospecimens that are routinely and prospectively collected in health promotion research (How Kit et al., 2012). Lastly, pursuing these new and feasible directions for using epigenetics in designing health promotion interventions and research will have implications for study design and methods. For example, epigenetic plasticity means that changes could be prompted by influences well beyond the scope of the intervention. Thus, in the situations of limited experimental control that characterize much of health promotion contexts, consideration of study designs (e.g., within-subjects designs) and comparison groups will be critical to enhance scientific rigor. Moreover, recent research highlights the importance of the aging process on epigenetic responses with some responses showing reverse associations in older and younger age groups (Sierra et al., 2015). Such effects could have design implications for intervention research including the composition of study samples.

## Potential applications of epigenetic concepts in health promotion interventions

In the context of common complex disease, we assert that epigenetic concepts could inform: (1) updates in health risk information that could improve the motivational potency of health communications, (2) the development of new approaches to targeted and tailored interventions, and (3) novel measures of intervention impact. In this section, we expand on these points, offering several examples of how epigenetics might inform the future of health promotion research (Table 1).

**Table 1:** Epigenetic discovery health promotion research innovation and translation

Epigenetic discovery	Health promotion research innovation domains	Example translational research questions
Individual variation in whether risk exposures negatively influence gene expression	Improving motivational potency of health communications	Evaluating relative benefit of validating beliefs about individual variability in extent of harm produced by risk behaviors on motivation
Intervention adherence can prompt measurable gene expression	Intervention targeting and tailoring	Comparative effectiveness of targeting relapse prevention approach based on gene expression profile following intervention participation
New technology to measure epigenetic processes (e.g., methylation) in saliva and blood samples	Novel biomarkers of intervention impact	Evaluate intervention adherence on gene expression in randomized effectiveness trials

### Improving motivational potency of health communications

Messaging is a key element of health promotion interventions with the challenge being how to frame health risk information in ways that motivate behavior change (Gallagher & Updegraff, 2012). New understandings of epigenetic processes have been suggested for use to update public health messages about health risks in ways that might enhance both their credibility and persuasiveness (Loi et al., 2013). For example, it is common to hear that the public is confused and frustrated by contradictory research findings related to risk exposures and health (e.g., alcohol intake is beneficial or harmful for some but not all health outcomes). Moreover, public health recommendations to limit risk behaviors can be inconsistent with direct experience—individuals are observed to stay healthy despite engaging in risk behaviors such as cigarette smoking or poor diet. This apparent lack of coherence in explanations could lead the public to question the validity of health recommendations (Cameron et al., 2012). There is strong conceptual support that the public’s explanations (or mental models) for individual differences in the health effects of exposures (e.g., poor diet, cigarette smoking) can influence their health behavior (Bostrom et al., 1992; Cameron et al., 2012).

Additionally, when asked about the causes of common health conditions, the public is likely to suggest health behaviors and genetics as key factors. Environmental exposures are less likely to be suggested, particularly by majority populations and those *not* living in social disadvantage (Robert & Booske, 2011). Thus many of those targeted by public health messages have limited imaginations for the role of social environment on health (Robert & Booske, 2011), and generally low literacy regarding how genes and environment *interact* to influence health outcomes (Condit & Shen, 2011). An example relevant to epigenetics is that some genetic susceptibility factors only become important in the presence of an environmental exposure. Given the large and persistent health disparities associated with social- and community-level exposures, improved understanding of genome responsivity to the environment could serve as a bridge linking social environment exposures to health outcomes, decrease victim blaming and galvanize public support for social environmental solutions to public health problems (Thayer & Kuzawa, 2011).

Health communications also could incorporate epigenetic concepts to explain how exposures such as lifestyle habits and the social environment can influence individuals differently. For example, descriptions of how accumulating exposures can turn genes on and off and influence health outcomes could be used to illustrate the need to make healthy lifestyle choices. Communications to increase understanding of environmental responsivity and life stages when risk might be heightened could be developed and evaluated to increase the salience of adopting risk reduction during those developmental stages. Explanations that validate beliefs about individual variability in response to risky lifestyle behaviors could be compared to general messages that recommend benefits for all, with regard to their relative influence on risk perceptions, motivation to reduce risk and behavior changes.

Among the many challenges these communication approaches will face is how to leverage mental models of individual variation in health outcomes while maintaining motivation and personal efficacy that risk reduction is needed and achievable. Communication strategies such as metaphors concerning environmental responsivity could be developed and rigorously evaluated for their effectiveness in reducing target audiences’ likelihood of ascribing a deterministic role to genetics (Cameron et al., 2012; Parrott & Smith, 2014). These principles will undoubtedly include conceptualizing ways that concepts relevant to epigenetics (e.g., risk uncertainty) can be applied to increase the motivational relevance and other constructs key to effective communications (Fischhoff & Davis, 2014). However an important caveat is that such communications will not have sufficient potency to promote behavior change (Hollands et al., 2016). Thus, research, guided by social and behavioral conceptual models, will be needed to evaluate whether these communication updates add value to evidence-based behavioral intervention approaches.

### Intervention targeting and tailoring

Emerging understandings of variation in epigenetic response also could be used to customize health promotion interventions. Consider the work of Crujeiras et al. (2013) that evaluated the association of epigenetic changes in specific genes with appetite control among men who had participated in a standard weight loss intervention (30 % calorie restriction goal). Compared to non-regainers, weight regainers (those who regained greater than 10 % of weight lost) were more likely to have genes involved in stimulating appetite turned on (i.e., lower total methylation at loci) and to have genes associated with appetite suppression turned off. These post-intervention epigenetic responses to weight loss could be used to tailor or target weight maintenance strategies to these groups. For example, interventions could emphasize prolonged ongoing support for those at highest risk and compare the value of these approaches to standard weight maintenance approaches.

Health promotion-related conceptual frameworks could be helpful for specifying individual and group-level exposures most germane for targeted or tailored interventions. Antonucci et al. (2014), for example, conceptualize accumulating exposures over a life span as a “convoy”. In considering the role of supportive others, they suggest that individuals acquire a convoy of relationships that move with them throughout the life course and change qualitatively over time. Linking this concept with epigenetic mechanisms suggests that health risks, and the success of health promotion interventions might be influenced by shifts in qualities of this convoy of support at windows of heightened responsivity. In considering which exposures are critical the researcher could pose questions such as what convoys of health behaviors, social support, or built environment exposures were occurring at responsivity milestones and have they changed detrimentally or beneficially over the life course? As well, such an approach could be used to identify those with exposure risk profiles and tailor or target interventions accordingly.

Such interventions might target groups who share “exposures” that occurred at important developmental junctures of high epigenetic responsivity (Mitchell et al., 2013). For example, intrauterine exposures have been shown to prompt epigenetic effects on neuroendocrine response and to be associated with increased likelihood of childhood and adult-onset obesity (El Hajj et al., 2014). Thus, obesity prevention interventions could be targeted to children born to obese mothers. Individually tailored interventions also could be evaluated as a motivational tool via personalized feedback to mothers regarding their child’s prenatal exposure. Integrating these approaches to leverage mother’s motivation to protect their children may be a particularly promising communication approach (Koehly et al., 2015). Each of these approaches have support from communication theory that they might increase the motivational relevance of health behaviors and prompt more thorough information processing than generic public health messages (Griffin et al., 1999).

### Novel measures of intervention impact

Understandings of epigenetics also could suggest new biomarkers that are more sensitive to intervention adherence and illuminate the processes through which interventions do or do not influence health outcomes. Too often large well-designed intervention trials that are based on strong conceptual models show null results, that is, no benefit of the intervention over comparison groups. Often intervention effectiveness is based on self-reported outcomes. Many researchers have raised concerns that the very act of completing repeated survey assessments could prompt behavior change among participants in comparison conditions or that responses reflect the heightened social desirability of reporting behavior change (DeMaio, 1985). Together, these factors may undermine the validity of self-reports, even when using rigorous behavioral assessments, and mask the benefits of health promotion interventions.

Health promotion research has a long tradition of using biomarkers (e.g., saliva samples) to validate self-reported behavior change where possible and to minimize related threats to validity when evaluating intervention effectiveness. Similarly epigenetic methylation processes could be assessed to indicate whether self-reported intervention adherence is concordant with physiological processes that might improve intervention adherence (e.g., release of dopamine associated with improved mood) or benefits of sustained behavior change (e.g., changes in gene expression associated with inflammation processes). These new approaches could give evidence of whether improvements in self-reported initiation and maintenance of behavior change deemed statistically *insignificant* are in fact concordant with physiological responses that suggest health benefit of intervention participation. It is possible that interventions shown to produce small improvements in health habits relative to a comparison group bring physiological benefits that are currently not being measured.

Bryan and colleagues are among the few research teams that evaluated the effect of participating in health promotion interventions and its association with epigenetic processes (Bryan et al., 2013). Their preliminary findings with 64 participants who participated in a 12-month exercise intervention gives insight into the link between physical activity and breast cancer. Self-reported physical activity based on the frequently used physical activity record (PAR) was associated with epigenetic modifications involved in turning off genes that prevent the cell proliferation that gives rise to malignant breast tumors. These epigenetic changes could be added as indicators of intervention benefit. Similarly, Ronn and colleagues assessed genome wide methylation in the adipose tissue of sedentary men before and after their participation in a 6-month exercise intervention (Ronn et al., 2013). The investigators analyzed abdominal adipose biopsies from men before and 48 h after their last exercise session. Results indicated a comprehensive increase in methylation (turning genes off) in all regions suggesting a more metabolically active adipose tissue after intervention participation.

In each of these instances, the health promotion researcher hypothesizes and tests, for example, whether adherence to a behavior change intervention is associated with methylation (appropriately turning genes off or on) that may be biologically beneficial for the health outcome of interest or influenced by intervention participation. Additionally, these approaches could enable evaluation of whether the intervention group or some subgroup of individuals based on intervention adherence level or convoy characteristics (e.g., life course social support or stress) show patterns of methylation consistent with a conceptual model or hypothesis. Thus, methylation patterns offer a measure of epigenetic modifications that may be more sensitive to intervention effectiveness.

## Epigenetic informed health promotion research: “How To” steps

Incorporating epigenetic-related biomarkers into health promotion interventions has been done relatively infrequently. However, those who have succeeded used a systematic approach that we have summarized in a three-step process. The process begins with development of a conceptual model that emphasizes the defined “exposome” most germane to the health outcome and target population (e.g., levels of influence as shown in Fig. 1). In subsequent steps, the model is used to guide selection of appropriate intervention components, and the genes and biological pathways that would be expected to be influenced by the intervention.

### Step 1: Settling on a conceptual model

Many have suggested that epigenetic processes can offer conceptual pathways to link levels of social and interpersonal influence on health outcomes (Burdge & Lillycrop, 2010; Loi et al., 2013; Thayer & Kuzawa, 2011). Though rarely operationalized beyond two levels of influence, the social-ecological framework now has a ubiquitous presence in health promotion research (Golden & Earp, 2012). Such multi-level conceptual models often are depicted as concentric circles of influence that are nested one within the other. These models favor comprehensive enumeration of all possible social and behavioral constructs of the “exposome” (Glass & McAtee, 2006). However, missing or only vaguely conceptualized in these complex depictions are the proposed mechanisms that connect levels of influence to health outcomes as depicted in Fig. 1.

An example is that most health promotion interventions achieve incomplete adherence. Poor adherence may be attributed to built environment factors (macro level), household factors (interpersonal level) and mood state (individual level). Thus, a focused social ecological model might be constructed using epigenetic mechanisms to link levels of influence to intervention adherence. For example, the conceptual model would consider epigenetic mechanisms (e.g., reward pathways) that may be prompted by or encourage intervention adherence within a specific context. In turn, inclusion of epigenetic assessment could illuminate whether, the intervention group or some subgroup of participants show patterns of methylation that vary in accordance with a multi-level conceptual model or hypothesis. Additionally, the model could posit potential effect moderators such as exposure convoys (e.g., changes in life course social support or stress) or adherence level.

Imagining potential methylation patterns requires some understanding of the families of genes that could plausibly be influenced by the intervention. This is important because the aim is not to evaluate all pathways but to parsimoniously consider those most plausible and specific to the influence levels (i.e., intrapersonal, interpersonal, social) under consideration. Lastly, a conceptual model can guide decisions about the appropriate comparison groups against which the influence of the intervention on epigenetic processes would be evaluated.

For example, Bryan et al. (2011) proposed a model in which cognitive and physiological pathways were suggested to enhance or diminish motivation to exercise and in turn, adherence to recommended activity level. In their model, genetic factors were hypothesized to influence mood benefit from exercise and perceptions of exertion. In turn, these factors were posited to jointly influence adherence to physical activity requirements. Lastly, adherence was thought to influence gene expression via epigenetic influences.

One limitation of Bryan et al. (2011) model is that these mechanisms were delineated only at a single level of influence (i.e., intrapersonal) without considering interpersonal or other environmental influences. One can imagine broadening this conceptualization to include co-exercisers, for example, who encourage (or ignore) each other during the exercise session as well as the availability of environmental opportunities to be physically active (e.g., access to recreational facilities). This broader conceptualization admittedly would be more complex. However, a conceptual framework could be constructed to narrow in on a socio-ecological “exposome” hypothesized to be most relevant for promoting physical activity that in turn, could guide hypothesis development regarding epigenetic mechanisms to consider.

### Step 2: Use the conceptual model to characterize appropriate intervention targets

Once the conceptual framework has been determined, the next step is to specify the intervention elements where influence could be identified via epigenetic mechanisms. Building on Bryan et al. (2011) example introduced above, the literature could support involvement at the interpersonal level of a co-exerciser as a means to improve adherence. The researcher would then consider through which mechanisms participants’ responses to this mutual support might influence activity adherence. Would it influence stress, making it easier or harder to adhere to activity recommendations? How might an exposure convoy (e.g., individual level or group shared exposures) influence the extent of these influences on activity? In turn, where would the effects of considering these exposures be expected to show up in downstream epigenetic processes associated with stress response? In this exercise, the researcher also must consider which intervention strategies to use to optimally influence adherence. Lastly, the choice of epigenetic assessments would be linked directly to the intervention approaches selected.

For example, Crujeiras et al. (2013) hypothesized that because energy balance is influenced by two competing mechanisms. Ghrelin secreted by the stomach that activates neuropeptides through epigenetic processes to stimulate appetite, and leptin secreted by fat cells suppresses the appetite via other epigenetic processes. These mechanisms suggest a testable hypothesis that an intervention tailored to the appetite-related methylation pathway might improve long-term maintenance of weight loss. Moreover, this approach suggested a new biomarker of impact, that is, could the maintenance strategy be linked directly to specific methylation patterns? As mentioned previously, those who regained more of their weight loss had methylation patterns indicating that genes associated with appetite control were turned off. These patterns were not present among those who maintained weight loss. Again, missing from this study was consideration of levels of influence beyond the individual. This could be accomplished by considering which higher levels of influence might also be influencing these methylation patterns.

### Step 3: Specify appropriate biomarker indicators to use as outcomes in evaluating the impact of the intervention

In this step, the researcher decides on the optimal epigenetic assessment and timing of measures. These considerations would logically build upon the prior steps in suggesting where and when epigenetic changes might occur if prescribed adherence levels were attained.

A prospective study conducted by Bryan et al. (2013) offers an excellent example of this process. The study evaluated whether self-reported increases in physical activity induced epigenetic patterns associated with reduced risk of breast cancer. To arrive at biomarkers, the authors reviewed work based on tumor cells of cancer patients versus controls to identify biologically plausible pathways through which increased exercise might reduce cancer risk. The authors settled on a gene-specific expression processes associated with cell death, “apoptosis-associated speck-like protein containing a caspase recruitment domain” or ASC. Epigenetic processes in which the ASC gene gets turned off, are associated with lower levels of inflammation. Inflammation in turn, has been consistently linked to cancer and other chronic diseases (e.g., obesity and Type 2 diabetes).

The researchers selected a priori the CpG islands linked to breast cancer acquisition and progression, and then developed a “composite measure” of these sites. Several sources were used to identify the epigenetic markers that comprised the composite measures. For example, genes and variants gleaned from a literature review included those studied among breast cancer patients taken from tumor cells and those suggested as possible preclinical markers for breast cancer. Other potential CpG sites were identified through an online annotation file available from Illumina; additional genes were identified that had been suggested to play a functional role in breast cancer development. In all, the researchers settled on 21 genes and 45 markers to analyze for their association with improvements in physical activity and hypothesized that DNA methylation across these sites would be negatively associated with self-reported physical activity levels (based on the PAR) and cardiovascular fitness (based on VO2 max). Saliva samples were collected prospectively at three-month intervals up to a year after intervention participation. DNA extracted from the saliva was analyzed via a commercially available Illumina platform that enabled methylation patterns to be evaluated (Bryan et al., 2013).

## Conclusions

Whether epigenetics can be used into improve health promotion interventions and research in the ways we have suggested raises numerous scientific questions worth exploring. A compelling advantage of pursuing this translational research is that emerging discovery in epigenetics may illuminate modifiable mechanisms that link different levels of influence on health and intervention benefits overlooked by current measures. Tractable research questions and related development of testable multi-level conceptual frameworks could move the field beyond the predominant focus of intervention research targeting a single level of influence. Accordingly, integrating epigenetics into health promotion research will call for intervention and methodological accommodations. Health promotion researchers can take the lead in keeping such research in the forefront of precision medicine discussions that will be increasing in the decade ahead. Indeed, the potential for launching a new generation of conceptual models, interventions and related methods informed by genomic discoveries should embolden us to gain the skills needed to engage in and advocate for this arena of translational research.
